# Enhancing Agency in Posttraumatic Stress Disorder Therapies Through Sensorimotor Technologies

**DOI:** 10.2196/58390

**Published:** 2024-07-01

**Authors:** Vladimir Adrien, Nicolas Bosc, Claire Peccia Galletto, Thomas Diot, Damien Claverie, Nicco Reggente, Marion Trousselard, Eric Bui, Thierry Baubet, Félix Schoeller

**Affiliations:** 1 Department of Infectious and Tropical Diseases Avicenne Hospital, AP-HP Université Sorbonne Paris Nord Bobigny France; 2 Institute of Psychiatry and Neuroscience of Paris Inserm UMR-S 1266 Université Paris Cité Paris France; 3 Department of Psychopathology Avicenne Hospital, AP-HP Université Sorbonne Paris Nord Bobigny France; 4 Unités René Diatkine Association de Santé Mentale du 13è arrondissement Paris France; 5 Department of Adult Psychiatry Impact, Mondor Hospital, AP-HP Université Paris-Est Créteil Créteil France; 6 Institut de Recherche Biomédicale des Armées Brétigny-sur-Orge France; 7 Institute for Advanced Consciousness Studies Santa Monica, CA United States; 8 INSPIIRE, Inserm UMR 1319 Université de Lorraine Nancy France; 9 ADES CNRS UMR 7268 Aix-Marseille Université Marseille France; 10 Department of Psychiatry Caen Normandy University Hospital Normandie Université Caen France; 11 Physiopathology and Imaging of Neurological Disorders UNICAEN, Inserm UMR-S 1237 Normandie Université Caen France; 12 Department of Psychiatry Massachusetts General Hospital Boston, MA United States; 13 Unité Transversale de Psychogénèse et Psychopathologie Université Sorbonne Paris Nord Villetaneuse France; 14 Centre National de Ressources et de Résilience Lille France; 15 Media Lab Massachusetts Institute of Technology Cambridge, MA United States

**Keywords:** posttraumatic stress disorder, PTSD, agency, proprioception, trauma, self-control, sensorimotor technology, enactive perspective, peritraumatic dissociation, proprioceptive reafferent fibers, gesture sonification devices

## Abstract

Posttraumatic stress disorder (PTSD) is a significant public health concern, with only a third of patients recovering within a year of treatment. While PTSD often disrupts the sense of body ownership and sense of agency (SA), attention to the SA in trauma has been lacking. This perspective paper explores the loss of the SA in PTSD and its relevance in the development of symptoms. Trauma is viewed as a breakdown of the SA, related to a freeze response, with peritraumatic dissociation increasing the risk of PTSD. Drawing from embodied cognition, we propose an enactive perspective of PTSD, suggesting therapies that restore the SA through direct engagement with the body and environment. We discuss the potential of agency-based therapies and innovative technologies such as gesture sonification, which translates body movements into sounds to enhance the SA. Gesture sonification offers a screen-free, noninvasive approach that could complement existing trauma-focused therapies. We emphasize the need for interdisciplinary collaboration and clinical research to further explore these approaches in preventing and treating PTSD.

## Introduction

Posttraumatic stress disorder (PTSD) stands among the 10 major public health issues [1]. Remission is obtained for a mere one-third of patients at 1-year follow-up, while another third continues to grapple with symptoms a decade later [2]. The gold standard treatments are trauma-focused cognitive behavioral therapies (CBTs), including cognitive processing therapy and prolonged exposure [3,4]. These approaches aim at improving self-regulation [5] but lead to remission in only approximately 40% of patients [6], although criteria for treatment nonresponse are not well defined [7].

Virtual reality exposure therapy (VRET) has been used with immersive simulations of trauma-relevant environments [8,9]. However, its superiority over standard prolonged exposure remains controversial [10-12], probably due to some inherent limitations, such as the sensory conflicts virtual reality (VR) induces as well as how its visual interface decouples patients from their body and environment [13]. In this context, other sensorimotor technologies letting patients connect to their environment may be of interest due to their potential to modulate the sense of body ownership (SO) and sense of agency (SA).

Emerging data have in fact reported how the SO and SA may be impacted in PTSD [14-19]. While the loss of the SO and control has been extensively studied in the context of PTSD and is historically among the main targets of trauma-focused therapies, much less attention has been paid to the loss and restoration of the SA, as opposed to emotion regulation and cognitive control. Despite promising theoretical perspectives on this topic under the umbrella of enactive theories of mind [15,20-28], there is still a dearth of agency-based treatment and recovery options available to patients with PTSD and clinicians. In this perspective paper, we explore the SA in the context of PTSD and its importance in understanding symptoms and improving treatments. Our contribution entails providing a road map for the development of agency-based therapies in the future, along with offering an agency-based perspective on psychological trauma. Finally, we explore the potential of using screen-free innovative technologies such as gesture sonification (GS) to complement existing trauma-focused therapies that target internal regulation by directly influencing the SA, thereby paving the way for the creation of new and effective intervention tools.

## SO and SA: Definition and Brief Review

The SO refers to the perception of one’s own body, feelings, thoughts, or movements, integrating somatosensory signals fundamental to distinguish between self and other, that is, to self-consciousness and control [29,30]. It depends on the interaction of afferent feedback and a top-down contribution of body representations [31,32]. The interplay between afferent internal (interoceptive and proprioceptive) and external multisensory (eg, visual and tactile) stimuli is sufficient for the SO [33], which is viewed as a psychophysiological baseline involving the brain’s default mode network (DMN) [34]. This multisensory integration in the premotor cortex enables bodily self-attribution [35,36], with the prioritization of the most relevant sensory domains [37]. Computational (Bayesian) models have been suggested to account comprehensively for this multisensory integration [38]. Conversely, the SA refers to the immediate feeling of initiating and controlling an action [39], that is, to the subjective perception of being an agent effecting changes in the external world with a sensorial reflection (ie, sensorimotor contingencies). That is, the SA refers to the feeling of being “the one who is causing some event in the external world” [40]. It depends on prior intention and prereflective perceptual monitoring of the consequences of self-generated action, achieved through basic efferent motor-control processes and sensory feedback [31,41-43]. The SA involves premotor, motor, and temporoparietal areas, intention, and action monitoring [34,44] and integrates sensory and motor signals into a coherent representation of the self-world system (ie, the sensory expectations deriving from motor output). The SA also relies on proprioception, which is broadly defined as the sense of (self-generated) movements, integrating signals arising from cutaneous, muscular, and joint receptors [45], and on causal attribution [46]. Therefore, the SA is best described as a multisensory process that integrates motor and nonmotor cues [47,48].

All in all, the SA is the sense of originating and controlling our actions, whereas the SO is the sense of being the one to act [29,39,47]. For instance, involuntary movement gives rise to the SO but not to the SA [42]. This phenomenological distinction has neural correlates [44], but recent work seems to show a more interactive model where the SA and SO are strongly related to one another [49-51]. For instance, both share a network in the left middle insula [52], and intereffector regions in the motor cortex display strong functional connectivity, suggesting that body control and action are part of a common circuit [53].

Prereflective SA and SO can be distinguished from attribution judgments [54-56], that is, the ability to attribute an action to its proper agent (self or other), which also integrates sensory information (the visual sense being determinant [57,58]). This integration processes multiple sources indirectly due to action and its congruence to the prediction of one’s self-narrative and understanding [41]. These second-order reflective (ie, cognitively processed) attributions of agency and ownership involve *judgments* of agency and ownership [42,47,59] and can be distinguished from first-order prereflective (ie, motor processed) *experience* (or *feeling*) of the SA and SO, although experience can influence judgment [60]. It is worth noting that this separation between the SO and attribution of ownership [61,62], as well as the separation between the SA and attribution of agency [63], has been challenged.

The SO can be measured by experiments, such as the rubber hand illusion (RHI) [33,35,64,65], in which a lifelike left rubber hand is viewed by participants whose real left hand is hidden, and the same afferent signals (eg, tactile stroke by a paintbrush) are being delivered to both hands, generating the transfer of the SO from the real to the rubber hand (ie, the illusion that the rubber hand is the real hand). Participants are then asked to point to their left hand with the right one and point toward a position between both hidden and rubber hands, elucidating the proprioceptive drift.

To measure the SA, both direct and indirect measures have been proposed [66]. Direct measures are rating scales or self-report questionnaires [66], whereas indirect measures focus on intentional binding [60], that is, the perceived time interval between the action and its outcome in comparison to involuntary action [67]. There are ongoing debates about the reliability and validity of these measures, as no correlations were identified between both types that may assess different aspects of the SA [68]. In this context, the Sense of Agency Scale, a new psychometric scale, has been developed and validated to directly assess global SA [69]. This self-report questionnaire includes 2 factors, the sense of positive agency and the sense of negative agency (respectively the control and lack of control over the environment), and is thus interesting for assessing altered SA in psychopathology. The Sense of Agency Scale has been validated in multiple languages [70]. The SA can also be measured in the laboratory with the RHI, which has been adapted recently [71] to a “dynamic” RHI where the index finger of the rubber hand moves when the participants move their own finger, both hands being mechanically connected, and the participants transfer their SA to the rubber hand (ie, feel being the ones tapping on the table with their finger). Interestingly, the illusion stays equally strong for various combinated sensory domains [72]. The SO and SA can be dissociated by varying the mode of movement (passive or active) and the position of the rubber hand (congruent or not). This strongly suggests that the SO and SA still represent distinct cognitive processes. The alteration of both SO and SA has been extensively studied across psychiatric conditions [42,57,58,73-75].

## Psychological Trauma as a Major Loss of the SA

Human experience constantly presents us with challenges that either meet our physical and cognitive skills or require that we develop new ones. When none of these are an option, or these options are overwhelmed, because of an unexpected event that goes largely beyond one’s representations and ability to adapt, threatens survival and physical or cognitive integrity of oneself or others [76], and induces a response of intense fear, helplessness, or horror, this event is called a *traumatic event* (TE; derived from *τραῦμα*, the Greek word for wound, hurt, or injury). In the Bayesian approach of the mind as a hierarchical predictive model of its reality, the TE does not relate to any predictive models available to make sense of the external world, as no empirical priors exist to account for the incoming sensory signals or the most likely motor response. Significantly, in severe cases of psychological trauma, the lack of a predictive model results in the loss of both vision and hearing [77]. From a physiological perspective, both central and peripheral regulatory systems are ineffective in carrying out their retrocontrol functions. The stress response system, which involves the activation of the amygdala at the central level and the corticotrope and noradrenergic hormonal systems at the peripheral level, is hyperactivated in contrast to the underactivation of brain structures responsible for typical retrocontrol [78,79]. This includes the prefrontal cortex (PFC) and the hippocampus [80-83]. Regarding circuit regulation, this aligns with an overactive salience network (SN; eg, amygdala with insula and the anterior cingulate cortex [ACC]), that is, with increased threat detection and fear learning [84], in contrast to weakly connected DMN and central executive network [85,86]. Along with the disruptions of the hypothalamic-pituitary-adrenal gland axis, the entire system is unable to effectively regulate the adaptive stress response.

Understanding the TE as a potential sensorimotor failure leads to a novel perspective of psychological trauma as *a major breakdown of the SA*. A lot of attention has been directed toward the SO and self-control, which depend largely on interoceptive signals (eg, “my body is unable to mitigate its own state of stress when being mugged”), whereas the clinical focus on the SA, directed toward exteroceptive signals, is insufficient. In the context of psychological trauma, the felt sense of helplessness (eg, “my body is unable to defend against an aggressor”), peritraumatic distress [87]; or tonic immobility, that is, the freeze response [88], translate into the failure of the SA. Hence, the breakdown of the SA automatically triggers a hardwired sensorimotor response, sometimes referred to as the defense cascade [88]. The alteration of both perception and action during the TE leads to dissociation [89], that is, disconnecting from incoming sensory information, generating a psychological distance from the traumatic experience, and allowing the patient to “tolerate” the intolerable [90]. Peritraumatic dissociation—an array of reactions to the TE that includes depersonalization, derealization, and emotional numbness [91]—has been thoroughly investigated as being the result of the absence of adequate sensory and motor representations (ie, “I do not understand what is happening to me and there is nothing I can do”). In derealization, the sense of the world is lost, whereas in depersonalization, the sense of self is lost. Emotional numbness corresponds to the absence of defensive emotions such as fear or anxiety. In certain cases, individuals may enter a state of sideration or extreme surprise, unable to move or plan any action, essentially losing their SA. Peritraumatic dissociation can be seen as a protective response to the intense emotional distress during the TE when there is no possibility of escape or avoidance and no sensory or motor representations of the event [23,92-94]. It provides a sense of safety and physical and psychic analgesia, reducing engagement with the TE [89,95]. Nevertheless and importantly, experiencing severe peritraumatic dissociation raises the risk of developing PTSD [96-99], interfering with trauma memory processing and coherence [89], thus, in this case, being deemed maladaptive, impeding the processing of the TE.

There is indeed a significant memory-related aspect of PTSD [100]: the emotions or sensory memories attached to the TE are either not integrated as long-term declarative memory, resulting in denial or the inability of verbalization of the TE, or are integrated as semantic instead of episodic memory [101,102], that is, as a factual general knowledge that does not seem to belong to the individual [103,104]. In other terms, the traumatic memory will be related to noetic instead of autonoetic consciousness [105,106]. This correlates with reduced activity or dysfunction in the hippocampal structures [107,108]. When the traumatic memory remains unprocessed, it fails to integrate with the individual’s conscious perception of reality. Over time, the failure to integrate the traumatic memory leads to a default in contextual processing [84,85,109,110], resulting in trauma-related cognitions, such as guilt and shame, which in turn induce a wide array of comorbid complications, such as depression [111], anxiety [112], and obsessive-compulsive disorder [113].

Posttraumatic dissociative states can also emerge later on as a protective reaction against the abnormal emotional associations linked to the traumatic memory [114]. Dissociative PTSD (D-PTSD) has been recognized in the *Diagnostic and Statistical Manual of Mental Disorders, Fifth Edition* (*DSM-5*) [115], and is supported by a neurophysiological basis that distinguishes it from conventional PTSD. Emotional overmodulation (ie, hypoarousal) in D-PTSD is thought to be linked to an underactivation of the amygdala and an overactivation of the PFC [114], in contrast to the standard PTSD model. The Clinician-Administered PTSD Scale for *DSM-5* [116] allows for differentiation between these 2 types of PTSD, and approximately 30% of individuals with PTSD may have the dissociative subtype [15,114,117]. It has already been demonstrated that patients with D-PTSD exhibit a higher proprioceptive drift in the RHI [15], suggesting that individuals with D-PTSD integrate the illusion more significantly due to their diminished SO. Further research is needed to determine if the SA is altered differently between these clinical subtypes. The latest version of the *International Classification of Diseases* [118-120] has recently introduced a subtype known as complex PTSD (C-PTSD), which is characterized by the symptoms of PTSD and 4 additional groups of symptoms: dissociation; difficulties in regulating emotions; a negative self-concept involving feelings of worthlessness, defeat, shame, and guilt; and challenges in social cognition, such as maintaining relationships and feeling emotionally close to others. This distinction is strongly supported by research [119,121,122]. C-PTSD affects approximately 40% of individuals with PTSD [123]. As for both D-PTSD and C-PTSD subtypes, there are currently no established guidelines for best practices.

Trauma-focused CBT may restore the functionality of the brain structures (PFC, ACC, and hippocampus) that are involved in executive retrocontrol, which is initially ineffective during the TE [124]. Notably, trauma-focused CBT is safe [125] and has been found to be as effective in treating D-PTSD [126,127], despite its reversed neurophysiological model, and C-PTSD [123] than conventional PTSD. By concentrating on the traumatic memory, the goal of this therapy is to alleviate the emotional dysregulation experienced with PTSD symptoms. Alternatively, the enactive perspective asserts that the traumatized brain is engaged, interconnected with the body and environment, and dynamically integrated [128].

## PTSD Symptoms Might Be Understood as an Attempt to Restore the SA

### Overview

When considering psychological trauma from an enactive perspective and acknowledging that perception and action are intrinsically linked, the mechanisms described in the previous section and their accompanying symptoms can also be understood as an attempt to control oneself, the environment, and the causes of the TE that pervade both perception and action, whether physically or virtually. Thus, understanding PTSD symptoms as an adaptive response to psychological trauma can inform future treatment development, creating a more holistic approach to PTSD treatment that focuses on empowering patients to regain an SA and control over their environment. In this section, we briefly review PTSD symptoms and how they relate to the SA (Table 1).

**Table 1:** Posttraumatic stress disorder (PTSD) symptoms, their relation to the sense of agency (SA), and how intervening on the SA may target these symptoms.

	How does the symptom relate to the SA?	How agency-based therapies can help?
Intrusion syndrome	Thoughts: attempt to get another shot at unfolding an optimal motor response to control the TE^a^. Repetitive behaviors: attempt to attribute to our own action the exposition or occurrence of the TE or the destruction of oneself during the TE.	Exposure therapy aims at self-control during intrusion symptoms, rather than the SA. Agency-based therapy may augment gradual exposure by letting the patient control exposure level and would aim at increasing environmental control and replacing repetitive behaviors by enhancing the SA.
Avoidance behaviors	Attempt to regain control over the environment and reminders of the TE or protect oneself from further harm.	Using gradual exposure therapy to triggers with body control of these triggers.
Hyperarousal and hypervigilance	Minimizes the expected loss of the SA (as a failure to avoid or control the TE, the patient anticipates it anytime).	The patient controls sensory triggers themself with the help of the therapist to regain adapted and coherent salience.
Trauma cognitions	Guilt: attempt to integrate the causes of the TE within a wider model of self, other, and reality in which oneself had some SA over what caused the TE to occur. Anxiety: signal anxiety, anticipate the expected loss of the SA, and work as a protection. Depression: loss of omnipotence or frustration of not having SA over the environment.	By controlling the environmental sensorial triggers, the patient can get pleasure in return, reducing anxiety and frustration and regaining some SA over the environment.
Dissociation	Loss of the SA. In addition, adaptive response against the passive loss of the SA felt during the TE or PTSD symptoms.	Increasing the sensorial signals produced by one’s action in the external environment reduces the disconnection felt between oneself and reality.

^a^TE: traumatic event.

### Intrusion Symptoms

Intrusive thoughts and memories as well as vivid re-experiencing, also called flashbacks, and traumatic nightmares are common symptoms in the aftermath of a TE [129,130]. These intrusive symptoms are distressing and make it difficult for patients to function in their daily life. Some patients with PTSD may have a tendency to replay the TE mentally in an obsessive-compulsive or addictive fashion, supposedly in an attempt to make sense of it and understand what had happened [131]. Intrusion symptoms can be regarded as a facet of dissociation [104]. Still, the recent findings on the activation of brain structures and neural circuits pointed out in the previous part, which would make PTSD and D-PTSD different entities, oppose this interpretation [114,132]. Intrusion symptoms have recently been analyzed from a Bayesian perspective, where the TE perceptual hypothesis gains a very high prior due to its life-threatening significance and is reselected independently of the actual sensory input [133]. From an enactive perspective, this can be understood as a way for the individual to try to regain some control over the TE to make it less distressing. Indeed, instructions to freely express intrusive thoughts led to a decrease in intrusive sexual assault thoughts over time, while suppressing them led to an automatic rebound in intrusive thoughts over time [134].

In addition to intrusive cognitions, repetitive behaviors, such as re-exposing oneself to potentially traumatic situations, are often seen in individuals with PTSD and can be a way for them to try to regain their SA. This can be observed in patients with C-PTSD and premorbid personality disorders, who may engage in risky or self-destructive behaviors [135]. Perhaps one of the most striking examples of repetition syndrome would be patterns of hypersexuality in children who have been victims of sexual abuse [136]. Patients are at risk of repeated harm, either self-inflicted or at the hands of others, adopting self-destructive behaviors [136] or repeated exposure to violence (suicide attempts, self-mutilation, military enlistment, development of substance use disorders, risky sexual behavior, and inability to give consent for sexual intercourse leading to exposure to rape situations), which can be seen as a tentative to attribute to our own action the destruction of oneself experienced during the original TE [121]. The climax of this viewpoint is found in the classical example of survivors becoming executioners (children who experienced abuse, child soldiers, etc) after a phase of repetitive behaviors such as engaging in plays or reenactments, often seen in childhood traumas [137]. Freud [138] famously discussed the idea of individuals using objects or activities to regain a sense of control over their environment. He gave the example of a baby playing with a toy that represents his mother to feel like he has control over the situation and soothe his distress. This idea was later developed by Winnicott [139] into the concept of transitional objects, used by children to cope with the loss of the subjective omnipotence over their environment, deriving from the caretaker’s attention to the child’s need, a frustration that can be related to the one experienced during a TE. Separation (ie, autonomy) from the parents accompanies the rise of the SA in childhood, as the sum of the attempts to compensate for the loss results in the gained ability to be alone. Intrusive cognitions and repetitive behaviors are thus common symptoms observed in individuals with PTSD, which can be seen as attempts to regain an SA and cope with the psychological trauma. However, these symptoms can also lead to repeated harm or victimization, making it crucial for therapists to develop treatment strategies that focus on restoring the SA while addressing the underlying causes of these symptoms.

### Avoidance Behaviors

The second category of PTSD symptoms is avoidance behaviors [140], that is, the active avoidance of thoughts, feelings, or external reminders of the TE. This can include avoiding certain places, people, or activities that may trigger memories of the event [141]. As patients have lost their SA over what triggers their flashbacks, the best way to control their occurrence is simply to avoid the stimulation. Here again, this might be understood as a displacement of the cause of the TE, where patients assume they can take responsibility for generating these sensory signals that largely surpassed their perception and action capabilities. One possible explanation for avoidance symptoms in PTSD is, therefore, that they may be an attempt by patients to regain their SA over their environment. For example, patients with PTSD due to a car accident may avoid driving, as driving may trigger memories of the TE [142].

An alternative explanation for avoidance symptoms in PTSD is that they serve as a means of self-protection, aiming to minimize the risk and vulnerability to risk. Social cognition impairment is commonly observed in PTSD [143]. By avoiding social situations, patients can shield themselves from re-experiencing the TE and encountering additional distress. Another instance is postdisaster PTSD, which is more likely to occur if the disaster originates from human actions as opposed to natural causes [144,145]. This underscores the greater psychological impact of interpersonal trauma, as it is perceived as potentially controllable, thus amplifying the distress. Patients may instinctively seek to regain the SA by avoiding situations associated with interpersonal trauma when addressing PTSD symptoms.

It should be noted that substance use is often seen as a form of avoidance in individuals with PTSD who may use drugs or alcohol as a way to numb or suppress their traumatic memories [146] or to escape from the difficult emotions that can accompany these memories. For example, self-reported PTSD is associated with increased use of 3,4-Methylenedioxymethamphetamine (MDMA) in adolescents with substance use disorders [147], and substance abuse is widespread in victims of childhood sexual abuse [148]. In addition to numbing their emotions, substance use can also be a way for individuals with PTSD to avoid trauma reminders (people, places, or situations).

### Negative Alterations in Cognition and Mood and Maladaptive Schemas

Negative cognitions are thoughts and beliefs about oneself, others, and the world that are negatively distorted and not based on reality. They include beliefs that one is to blame for the TE, that the world is dangerous and unpredictable, and that one is not safe. They also include decreased interest in activities, negative emotions, and the feeling of being isolated. They may be a way of coping with the loss of the SA. Patients with PTSD often believe they are to blame (ie, the feeling of guilt) for the TE, perhaps as this belief provides them with a (virtual) sense of control over the situation. By experiencing guilt and self-blaming, they make sense of the event and feel like they have some SA over it—“I am responsible for exposing myself to the environment that generated the TE” [149].

In addition, negative cognitions in PTSD relate to the prediction of an *expected* loss of the SA. A TE can cause a person to feel vulnerable and at risk: negative cognitions may be a way of anticipating and preparing for future loss of control. For example, patients with PTSD may believe that the world is dangerous and unpredictable, as this belief helps them to be prepared for potential threats. This culminates with the concept of signal anxiety developed by Freud [150], one of the main defense mechanisms. By expecting the worst, patients are able to feel like they have some control over their environment and can protect themselves from further harm. Klein [151] related trauma negative cognitions to the “depressive position” in the early phase of existence. This preverbal infantile attitude comes with a loss of subjective omnipotence (ie, control) over external objects that coincides with the emergence of emotional valence in infancy: the child becomes aware that objects not only gratify (“good object”) but can also frustrate (“bad object”), generating feelings of guilt and grief and a desire for reparation. This example of “proto-traumas” or “micro-traumas” experienced repeatedly during early childhood lightens what happens when a real TE occurs later in life, altering the sense of control and leading to greater PTSD susceptibility [99,152,153]. These negative thoughts and beliefs may be a way of coping with the distress and vulnerability caused by the TE, but in a vicious cycle, they can also have negative effects on daily life and overall well-being related to an expected loss of the SA, if nothing is done to remedy it.

### Alterations in Arousal and Reactivity: Disturbed Attentional Patterns

Patients with PTSD develop a heightened state of awareness, alertness, and physiological arousal to regain a sense of control and SA [154]. This is referred to as hypervigilance [114], characterized by emotional undermodulation, resulting in an increased threat detection and fear learning, and a state of alertness and readiness to respond to these threats [155]. This also includes irritability, difficulty concentrating, sleeping disorders, and an overall sense of feeling on edge. On a sensorimotor level, hypervigilance may manifest as an increased sensitivity to sensory stimuli, such as changes in light or sound, as well as increased physiological arousal, such as increased muscle tension or an elevated heart rate, which is a risk factor for developing PTSD when it immediately follows the TE [156,157]. PTSD is in return a risk factor and shares genetic risk for cardiovascular diseases [158]. By being constantly on guard and ready to respond to potential threats, patients may feel they are able to anticipate and prevent dangerous situations from occurring. This restores the SA that was lost as a result of the TE. Hypervigilance, as an attentional bias toward potential threats, can lead to anxiety and further hypervigilance [155,159]. This can cause the individual to misinterpret ambiguous cues as threats and exaggerate minor threats, as shown by the overactive SN, whose function is to recognize and prioritize stimuli while regulating emotional reactivity [160]. These changes in attention and threat perception can also manifest in the individual’s eye movements [161], leading to an inability to disengage from potential threats. This is referred to in the literature as “oculomotor reflexes” [162]. From a Bayesian inference perspective, dysfunctional SN amounts to aberrant precision control, where precision denotes the confidence placed in prediction errors (mirroring the reliability of the stimulation that causes them) within the hierarchy of information processing, that is, a high precision will favor bottom-up ascending prediction errors, while a low precision will bias perception toward top-down prior beliefs. Interestingly, the SN implies the ACC and insula, which are strongly related to interoception [163]. The dysregulation of bodily signals in psychiatric illnesses may, therefore, offer an important way forward in terms of phenotyping [164,165].

### Dissociative Symptoms

After Winnicott [139], Anzieu [166] developed the concept of “skin-ego,” that is, the presence of a symbolic skin allowing for the creation of psychic and bodily boundaries. In PTSD, this symbolic frontier is blurred or constricted and no longer filters or organizes sensory and perceptual signals as easily. It becomes porous and loses its function as a protective structure between interoceptive cues and cues originating from the external world. This sudden immense vulnerability exacerbates the loss of self-integrity and SO and forces the maintenance of a state of psychic survival with various defensive symptoms involving both temporality and space: agitation, withdrawal, and dissociative symptoms.

The view of peritraumatic dissociation as a protective response can be related to the anthropological interpretation of dissociative experiences with their positive affective valence, with the DMN mainly activated in situations such as daydreaming, hypnotic responses, fatigue, anxiety, drug intoxication, and boredom [167]*.* In this case, dissociation has a social and discursive meaning rather than being seen as a mechanism. It can thus function adaptively, which depends fundamentally on context [94,168].

By contrast, dissociation interferes with the integration of sensory information and the creation of a coherent sense of self. If dissociative symptoms of PTSD are the reminiscences of peritraumatic dissociation and protect patients against the distress provoked by intrusion symptoms or hyperarousal, they are nevertheless severely debilitating. Patients often complain of the distress related to their dissociative states, which can be transitory or permanent and are associated with high disability. Dissociation can indeed be interpreted literally as the loss of the SA: “if I see myself from an external point of view, thus my body’s actions do not belong to myself” and “if the external world is not real anymore, then I can no longer be an agent in the real world.”

Dissociation can thus at the same time be a loss of the SA and a protective phenomenon against it. There is an ongoing debate on this matter, with the concept of protective dissociation culminating in at-risk professions such as the military or fire service: workers experience deliberate dissociative states [169,170] to gain automatic action and execute tasks more efficiently without being emotionally overwhelmed. Further research is required to thoroughly investigate and clarify this phenomenon. One potential avenue is to distinguish between the *passive* loss of the SA resulting from external TE and the symptoms actively produced (deliberately or not) by patients as a means to combat passivity (“if it is due to my action or my mind that I lose SA, thus the weight of external events in the loss of SA is reduced”). This includes dissociation or dissociative behaviors (eg, drug use).

## Sensorimotor Technologies for Agency-Based Therapies

The enactive, agency-based perspective on PTSD suggests that the disruption of the SA results from the TE, overwhelming the patient’s ability to adapt and make sense of sensorimotor signals, leading to a breakdown in the hierarchical predictive model of reality. This disrupted SA may contribute to or be further exacerbated by peritraumatic dissociation, explaining why its intensity is a predictive factor of PTSD onset [96-99].

Currently, there is a lack of agency-based treatment options for PTSD. New treatment options to manage nonresponse to PTSD conventional treatments [7] include the promising use of ketamine [171] or psychedelic-assisted psychotherapy [172], whose dissociative effect is now reconsidered as a phenomenological therapeutic tool, as these changes of the self-experience have a subjective meaning and are transitory and necessary for coping. These drugs could help improve prefrontal function and contextual processing, modifying beliefs, refining predictions and thus the SA [173-178]. Ketamine could also induce brain-derived neurotrophic factor increase in the hippocampus [179]. Conversely, endocannabinoids modulators or drugs that reduce the glutamate response (such as D-cycloserine, a partial N-methyl-D-aspartate [NMDA] receptor agonist) could not only treat dissociative PTSD symptoms [180,181] but also enhance extinction training by preventing the hyperglutamatergic state of the stress response responsible for abnormal fear conditioning [182,183] as well as the defaults in memory processing [181]. Indeed, NMDA receptors are highly concentrated in the hippocampus and implied in long-term potentiation, a mechanism for encoding long-term (eg, episodic) memory. Neuromodulatory treatments [7] such as transcranial magnetic stimulation [184-186], transcranial direct current stimulation [187,188], deep brain stimulation [189], and vagus nerve stimulation [190,191] could increase body awareness, SO, and SA. Nevertheless, less invasive or expensive treatments, such as sensorimotor therapies [192-194], may have the potential to enhance and restore the SO and, more importantly, SA during PTSD recovery. A mounting body of evidence [165] suggests that bodily signals play an essential role in driving precision control, hinting toward the relevance of reliable body-based interventions for mental health disorders depending on the patient’s life history, conditions, and symptoms. The controlled generation of artificial sensations could, therefore, lead to novel options for the diagnosis, monitoring, intervention, and treatment of disorders of emotional and interoceptive inference. One such potential solution is “*affordance training*” with a narrative therapy map [195], which involves helping the individual to focus on action possibilities rather than action impossibilities. This can involve training patients to engage in goal-directed behavior and to seek out opportunities for action, rather than focusing solely on potential threats.

Sensorimotor technologies, such as VR, have been used for exposure therapy in immersive simulations of trauma-relevant environments [8,9]. The aim is to allow a precise control of stimulus [196]. VRET can discriminate between patients with PTSD and patients without PTSD on a measure of psychophysiological arousal such as skin conductance reactivity [197,198] as well as between patients with low symptoms of subthreshold PTSD and patients with high symptoms of subthreshold PTSD through heart rate [199].

However, various meta-analyses did not find any difference in clinical efficacy between conventional VRET and other psychotherapies [10-12], although some found moderate effects [200]. However, specific VR-graded exposure therapy seems to be beneficial [11]. All in all, VRET may be beneficial only for specific patient profiles (eg, those who are younger, with greater hyperarousal symptoms, with comorbid depression or suicidal risk, with no antidepressant medication, or who cannot engage in imaginal imagery) compared to prolonged exposure therapy [201-203]. One explanation of the limitation of VRET is that patients may become diverted by the technology, expressing doubts about its authenticity (“this isn’t real”) and using this divergence or the lack of personalization of virtual environments as a means to evade forming an emotional connection with their distressing memories [196], especially because sensorial integration prioritizes sight, as shown by the RHI [65]. Indeed, VR deprives patients of their gaze and hands due to the screen and interface, creating a disconnection between the patient’s body and its environment, a restriction of virtual objects as actionable targets, contrary to tangible real-world objects [204], compromising the transfer of visuomotor skills from virtual to real settings [205]. n addition, the disembodying effect of VR can create interoceptive and visuo-proprioceptive issues that make the experience less immersive [206,207], besides causing discomfort, headaches, nausea, or instability [205,208]. Another issue is the lack of facial or trauma-focused interaction with the therapist, which can be problematic for the therapeutic alliance, limiting a shared experience, impeding regaining a sense of social safety [209-211] as well as the possibility of group therapy, a useful method in PTSD [212]. In this context, other sensorimotor technologies letting patients connect to their environment may be of interest as an alternative or adjunctive intervention. They could provide individuals with an SA over their environment.

## The Example of GS Technologies to Treat PTSD

### Overview

**Figure 1:**
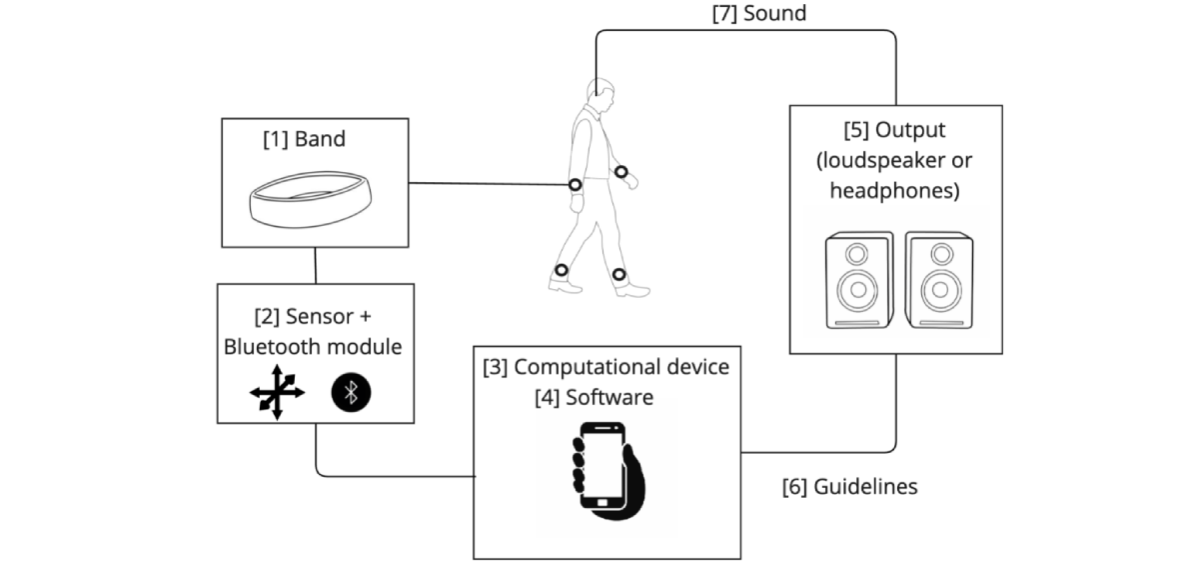
System diagram of gesture sonification illustrating (1) the integration of a wearable band (2) with sensors and a Bluetooth module, (3) which communicates with a computational device (4) running specific signal processing software. The device (5) processes the data through loudspeakers or headphones (6) following the therapist guidelines (7) to provide auditory feedback.

GS is a technique that involves using natural body gestures to control and generate sound. It uses sensors to detect the movement of an individual’s body and then translates this movement into sound (Figure 1).

GS allows overcoming VR’s limitations, as it is a screen-free alternative where only the auditory sense is augmented. It enables participant-body-environment coupling, the maintenance of contact with the therapist, and a more individualized virtual environment. It is applicable in case of language barrier, is more acceptable and user-friendly, and provides clinicians with ample opportunities for exploration. However, despite its potential benefits, some challenges might be encountered, such as acceptability. Indeed, patients may refuse to use GS; feel inhibited or startled while using it; and be exposed to various risks, such as allergies to sensors or, in case of improper use, hearing loss due to excessive volume or physical injury from uncontrolled movement. In addition, it is important to exercise caution both in the moment and in selecting sounds according to the way GS is used. For instance, some sounds that might be used for providing a sense of security in some patients could trigger intrusive symptoms in others due to specific traumatic experiences (eg, water sounds for a patient who experienced drowning).

While GS has found applications in various health contexts, such as physical rehabilitation [213] and music therapy [214], its potential in psychiatric illnesses has largely remained untapped. By combining GS with sensorimotor exercises, patients with PTSD could use their bodily movements to generate sounds and manipulate and control virtual objects and environments, creating a more immersive and engaging experience that leaves room for interaction with the therapist. GS could, therefore, emphasize the body and the sensations of movements that are often overlooked in first-line therapies such as prolonged exposure. By directing attention to the body and the physical sensations experienced, GS could assist patients in connecting with their present moment experience and developing a greater SA [193,215]. Indeed, multisensory retroaction can reinforce patients’ sense of control over their symptoms [213]. Importantly, GS can be combined with guided imagery (GI) to provide a personalized experience for patients, allowing them to manipulate, through sensorimotor exercises, the traumatic memories and facilitate their integration. GI is a behavioral mind-body intervention using appropriate scripts and imagination to manipulate representations and enable positive affective and body responses [216,217]. GI uses sensory integration to enhance affective and cognitive retrocontrol over hyperarousal through muscular relaxation and positive mental images. GI has been shown to improve depression, anxiety, and stress [218] and change the meaning of pain [219] and is also widely used in prolonged exposure as well as a self-management intervention to alleviate PTSD symptoms [220], especially hyperarousal. Nevertheless, individuals are not equal in their ability to engage in GI, and its efficacy is correlated with absorption abilities [221] and stays controversial [222]. Our team has experimented with GS and found that the technology could potentially help augment such abilities and thus mind-body interventions. We identified 5 potential applications of the system following the common course of existing therapies.

### PTSD Primary Prevention

Functional alterations in brain circuitry identified in patients with PTSD could already be present before the TE and constitute risk factors for developing PTSD [83]. Occasionally, they may exist before PTSD and worsen after the onset of PTSD. Indeed, PTSD has been found to be highly heritable due to epigenetic factors [83,223-226], which means that prior trauma and cumulative life adversity may induce alterations in brain circuitry responsible for vulnerability to PTSD [99,152,153].

Preventing PTSD, for instance, among at-risk populations such as women, refugees, and military and rescue workers [227] involves more than just averting TEs. It means identifying cognitive impairments predisposing for PTSD and enhancing or correcting these impairments. This includes improving cortical and hippocampus retrocontrol abilities, activating the central executive network or DMN, and regulating the SN to maintain threat detection at an optimal level. Ultimately, this entails bolstering the SA before the TE occurs, potentially averting SA failure, peritraumatic dissociation, and thus PTSD later on. GS could be used to assess vulnerability and prepare military and rescue workers before field operations.

In addition, GS could serve as an SA enhancer in the immediate and postimmediate phases following the TE. During the immediate phase, where defusing by talking [228] aims at reintegrating individuals in the present moment with the presence of the other, GS could help mitigate peritraumatic dissociation by amplifying environmental sounds and facilitating reorientation. In the postimmediate phase, during early psychological interventions or debriefing, which may not prevent the onset of PTSD but may improve subsequent adherence to necessary care [229-235], GS could help reduce stress activation by providing soothing sensorimotor environments.

### Reducing Hyperarousal and Negative Alteration of Mood: Securitization

GS could potentially help patients with PTSD to reduce their hyperarousal symptoms. Patients may indeed engage their senses to focus on the present moment. Using personalized sound environments considered secure by patients (eg, water, beach, and fireplace) can help reduce physiological arousal (eg, elevated heart rate or increased muscle tension). Furthermore, by letting patients control with their body movements the volume of the sounds produced by the device, it may help them acquire the ability to filter the external sensory signals whose input is distorted in PTSD with hypervigilance.

By strengthening proprioception and sensory modality with movement, patients can learn to tame the external world, the one that has betrayed or shattered. GS would thus help construct coherence and new congruences in multisensory processing: sorting, filtering, or hierarchization. Enhancing the patient’s self with an auditory signal, a vibrational charge to movement from the environment, offers the patient the opportunity to reverse defensive modes of withdrawal: it awakens curiosity and momentarily diverts anxious rumination about the external world. The environment becomes a facilitator of action, contributing to the reshaping of mental images and the reinforcement of motor planning. The patient actively participates, seeking to recombine the missing pattern in psychological trauma, creating sensory coherence and ideas through action. This synergistic process involving the postural system and the psychomotor feedback allowed by GS nourishes past sensorimotor experiences while updating new ones. Patients are placed in a position to synchronize with the world and derive pleasure from this tuning.

### Reducing Dissociation: Reassociation of Patients With Their Body

Importantly, GS could help patients with PTSD to reduce dissociation symptoms. Sensorimotor therapy facilitates the reconstruction of the body schema to align with functional reality. Patients are guided to inhabit their body and reshape their representations within a somatic reality. Through the pursuit of body wholeness and conscious work on muscle tone, the therapist contributes to this aim. Dance, slow movement in dynamic relaxation, and postural work (eg, Qi gong and yoga), through the antagonistic interplay of tension and relaxation, create a dynamic musculo-psychological synergy. Tonus is initially a function of dialogue with the world. Among the wide variety of sensorimotor or body-oriented therapies, GS could be combined with dance movement therapy (DMT) for instance. DMT has demonstrated its effectiveness in treating PTSD [236,237]. It calls for the awakening of supportive responses: a confident relationship with support generates a repelling force. Attention is focused on coordination and responses to orientation, alignment, and balance in rhythm. DMT is a therapeutic mediation that allows for the revisiting of early tuning connections; engages in the quality of gaze, joint attention, and gesturing; and addresses support, verticalization, and initial connections. Combined with GS, patients may be able to control with their body various elements (eg, rhythm, timbre, melody, harmony, and dynamics) of the music to which they are dancing and position themselves in a realm beneath language: the reality of music interplaying with movement that spatializes and shifts. This dual modality is a prelude to the transformation of psychological trauma through somato-psychological adjustments, when the body becomes “the thought-thinking apparatus” [238]. In essence, GS combined with sensorimotor therapies would work toward the generation of new body images, reinforcing tonic regulation, self-esteem, and primary narcissism and finally restoring not only proprioceptive feelings enabling patients to reintegrate their body, as well as a sense of contact to the ground (ie, reducing depersonalization), but also the SA over incoming sensory signals from external objects (ie, reducing derealization).

### Reducing Intrusion Symptoms and Avoidance: Exposure to the TE

GS involving sounds related to triggers of intrusive symptoms or to the TE itself may help patients with PTSD to engage with their environment in a more active, meaningful way than mere passive exposure, controlling intrusive symptoms and overcoming avoidance behaviors. Gradually, they would expose themselves to triggers that they are avoiding, gaining progressively the ability to control them. GS may indeed let the patient control the level of gradual exposure to TE triggers by themself. As in prolonged exposure, the uncontrollability of intrusion symptoms or triggers would then be reduced by the gained ability to control the irruption, intensity, and end of triggers or intrusion symptoms through body-environment interactions.

### Social and Cognitive Rehabilitation

Social cognition is disturbed in PTSD [143], especially social perception, affective theory of mind, affective empathy, and social interactions. Of note, social cognition is more altered if the TE is interpersonal (ie, originating from human actions) than not interpersonal [239]. Patients with PTSD exhibit an overactive SN, which has a crucial function in affective empathy, which in turn also relates to a higher sensitivity to stress [240] or the negative social impact [241]. The potential higher affective empathy may probably explain the increasing attention on social rehabilitation in PTSD treatment, such as communication training, group therapies, or community programs [242]. Indeed, social rehabilitation favors the sense of belonging, just as collective commemorations of disruptive events are perceived useful by victims and help rebuild social links [243].

Sensorimotor group therapies have recently been found to be effective in treating C-PTSD [244,245]. Just as GS would help restore localization and a sense of contact with the ground and external objects by increasing the sensorimotor afferences, it could also restore the sense of contact with other individuals and help in the process of mourning. The involvement of the body and the sensorimotor pleasures that reconstruct and reshape body schemas enhance bodily sensations of gathering or synchronization. This, in turn, boosts the effective connectivity of the mirror neuron system, which is crucial for social cognition [246].

## Conclusions

In this paper, we offer a perspective of PTSD in the light of the essential role of the SA in functional behavior. Recognizing the TE as a sensorimotor failure, we view psychotrauma as a profound breakdown of the SA. We offer an enactive perspective of PTSD, where symptoms represent efforts to uncover and restore the SA in response to the TE. We advocate for PTSD therapy to develop interventions fostering direct engagement with one’s body and environment, gradually rebuilding the SA. We suggest that agency-based therapies could mitigate PTSD risk and enhance treatment effectiveness. GS, alongside active somatic or movement-based approaches such as sensorimotor therapy, DMT, and somatic experiencing, directs attention to the body and sensory experiences, fostering present moment connection and bolstering the SA. Overall, this paper highlights the significance of the SA in psychotrauma and PTSD, offering insights to enhance treatments and advocating for further research in this critical area.

## Abbreviations

ACC: anterior cingulate cortex
CBT: cognitive behavioral therapy
C-PTSD: complex posttraumatic stress disorder
DMN: default mode network
DMT: dance movement therapy
D-PTSD: dissociative posttraumatic stress disorder
GI: guided imagery
GS: gesture sonification
MDMA: 3,4-Methylenedioxymethamphetamine
NMDA: N-methyl-D-aspartate
PFC: prefrontal cortex
PTSD: posttraumatic stress disorder
RHI: rubber hand illusion
SA: sense of agency
SN: salience network
SO: sense of body ownership
TE: traumatic event
VR: virtual reality
VRET: virtual reality exposure therapy
